# Why a Complete Response Is the Treatment Aim in Chronic Spontaneous Urticaria

**DOI:** 10.3390/jcm12103561

**Published:** 2023-05-19

**Authors:** Jonathan A. Bernstein, Ana Giménez-Arnau, Marcus Maurer, Petra Staubach, Nathalie Barbier, Eva Hua, Thomas Severin, Pedro A. Laires, Maria-Magdalena Balp

**Affiliations:** 1 Division of Rheumatology, Bernstein Allergy Group and Clinical Research Center, College of Medicine, University of Cincinnati, Cincinnati, OH 45267, USA; 2 Dermatology Department, Hospital del Mar, IMIM, Universitat Pompeu Fabra, 08005 Barcelona, Spain; anamariagimenezarnau@gmail.com; 3 Institute of Allergology, Charité–Universitätsmedizin Berlin, Corporate Member of Freie Universität Berlin, Humboldt-Universität zu Berlin, 12203 Berlin, Germany; marcus.maurer@charite.de; 4 Fraunhofer Institute for Translational Medicine and Pharmacology ITMP, Allergology and Immunology, 12203 Berlin, Germany; 5 Department of Dermatology, University Medical Center Mainz, 55131 Mainz, Germany; 6 Novartis Pharma AG, 4002 Basel, Switzerland; 7 China Novartis Institutes for Biomedical Research Co., Ltd., Shanghai 201203, China; 8 National School of Public Health, Public Health Research Center, Universidade NOVA de Lisboa, 1099-085 Lisbon, Portugal

**Keywords:** chronic spontaneous urticaria, complete response, patient-reported outcomes, quality of life, sleep

## Abstract

This study investigated the association between urticaria activity and health-related quality of life (HRQoL). Patient evaluations from the ligelizumab Phase 2b clinical trial (N = 382) were pooled (NCT02477332). Daily patient diaries assessed urticaria activity, sleep and activity interference, the dermatology life quality index (DLQI), and work productivity and activity impairment-chronic urticaria (WPAI-CU). The number of DLQI scores, weekly sleep interference scores (SIS7), weekly activity interference scores (AIS7), and overall work impairment (OWI) evaluations with a complete response per weekly urticaria activity score (UAS7) using bands (0, 1–6, 7–15, 16–27, and 28–42) were reported. Over 50% of the patients had a mean DLQI of > 10 at baseline, indicating a significant effect of chronic spontaneous urticaria (CSU) on their HRQoL. Complete response (UAS7 = 0) evaluations corresponded with no impacts on other patient-reported outcomes. In total, 91.1% of UAS7 = 0 evaluations corresponded to DLQI scores of 0–1, 99.7% to SIS7 scores of 0, 99.7% to AIS7 scores of 0, and 85.3% to OWI scores of 0. This was significantly different compared with the UAS7 = 1–6 evaluations (61.9%, 68.5%, 67.7%, and 65.4%, respectively; *p* < 0.0001). Complete responses to treatment were associated with no impairments on the dermatology-QoL, no interferences with sleep and activity, and significantly improved capacities to work compared to patients who continued to have signs and symptoms, even for those with minimal disease activity.

## 1. Introduction

Chronic spontaneous urticaria (CSU) presents predominantly on the skin, with clinical signs and symptoms such as itching, hives, and angioedema. The signs and symptoms of CSU can reoccur for more than 6 weeks [1], and the hives and itching can last from a few hours to a few days, with or without angioedema. The pathophysiology of CSU involves mast cells as key drivers. Mast cell degranulation results in the release of proinflammatory mediators including histamines that induce vasodilation and increase extravasation, itching, and the recruitment of inflammatory cells to lesions. Mast cell activation in CSU is driven by several mechanisms. One of the prime mechanisms involves immunoglobulin E (IgE), either directly or indirectly (IgE autoantibodies, anti-IgE, and anti-FceRI) [2]. Ligelizumab and omalizumab are monoclonal anti-IgE antibodies that neutralise IgE, and thereby, they prevent its binding to mast cell surface receptors.

The negative impacts of CSU symptoms significantly affect many aspects of patients’ health-related quality of life (HRQoL) [1,2,3,4,5]. The unpredictability of attacks can lead to patients feeling a loss of control over their lives [6], adding to the overall burden of the disease. The assessment of disease activity using only wheal numbers, itch severity, or angioedema does not provide a comprehensive account of the disease burden. Patients have described restrictions on their social life, feeling embarrassed, sexual problems [7], effects on their mental health [8], sleep interference [9], and the inability to work as some of the major aspects that affect their HRQoL [3,4,10]. 

Clinical studies and real world evidence have shown that patients with CSU experience a significant effect on their capacity to work; in fact, over 20% of patients have reported at least 1 h of missed work per week, with an overall work impairment (OWI) of 27% [4], and this impairment increases with greater disease activity severity [11]. CSU is also associated with sleep interference, including difficulties with falling asleep and awakenings during the night [12]. Lack of sleep can, in turn, lead to a poor HRQoL, especially when wellbeing is further impaired by physical signs such as angioedema [13]. In patients with poorly controlled CSU, the incidence of sleep interference is almost double that reported for age- and sex-matched controls [14].

Evidence has shown that the level of disease activity has a significant impact on patients’ HRQoL scores and sleep [11,15,16,17]. There appear to be significant differences in terms of disease burden between various weekly urticaria activity score (UAS7) disease states [17]. Furthermore, there is a high correlation between changes in disease activity and dermatology-related quality of life (QoL) [17]. Continuous evidence is required to demonstrate the true humanistic and economic value of achieving a complete response to treatment and to ensure all clinicians recognise this; this is the key recommendation of the international EAACI/GA^2^LEN/EuroGuiDerm/APAAACI guideline for urticaria—to treat the disease until it is fully resolved [18]. 

CSU is a complex disease with several interlinked aspects, including disease activity, impact and control, that are assessed using different tools [19,20]. By using the appropriate tools, physicians aim to determine the effects that urticaria has on a patient’s daily life, such as effects on their QoL, sleep, daily activities, and work. Patient-reported outcome measures (PROMs) are important tools that assess a patient’s perspectives on the actual burden of disease and capture treatment effects that help to contribute to the optimisation of disease management [21]. Despite the important insights that PROMs offer to physicians about the individual dimensions of a patient’s life, there is limited information on how these different dimensions measured by PROMs are linked, for example, how a patient’s CSU disease activity PROM score impairs their dermatology-QoL and/or work productivity PROM scores. A holistic understanding of these associations would optimise the usefulness of PROMs and enable physicians to plan appropriate treatment strategies. This is now even more important considering the key recommendation to treat the disease until it is resolved [18]. The objective of this analysis was to assess what impact disease control has on other aspects of a patient’s life, such as dermatology-QoL, sleep, daily activities, and work, from a patient-reported outcomes perspective.

## 2. Materials and Methods

### 2.1. Study Design and Patients

Data from the Phase 2b clinical trial of ligelizumab for CSU were used for this analysis [2]. Briefly, this randomised, double-blinded, active- and placebo-controlled study included adult patients with moderate to severe CSU (defined by a UAS7 score of ≥ 16 on a scale from 0 to 42) who were inadequately controlled by H_1_-antihistamines. This analysis included all 382 patients in the study who were randomised to receive ligelizumab (24 mg (n = 43), 72 mg (n = 84), 120 mg (single-dose arm, n = 42), and 240 mg (n = 85)), omalizumab (300 mg (n = 85)), or a placebo (n = 43). The patients received treatment every 4 weeks, with a total of five injections over 20 weeks, and they had a treatment-free follow-up period of up to 24 weeks (NCT02477332).

### 2.2. Assessment of PROMs

Patients completed the urticaria patient daily diary every day during the study, comprising the twice-daily UAS, itch severity score (ISS), and hives severity score (HSS), and they recorded their sleep interference scores (every morning) and levels of activity interference (every evening) [22,23]. Weekly scores for each concept were calculated as the sums of daily scores over 7 days. The UAS7 is the guideline-recommended gold standard for measuring disease activity in patients with CSU [24]. UAS7 disease activity scores are defined using the following categories: complete response (UAS7 score = 0), well-controlled activity (UAS7 score = 1–6), mild activity (UAS7 score = 7–15), moderate activity (UAS7 score = 16–27), and severe activity (UAS7 score = 28–42) [17]. The weekly sleep interference scores (SIS7 score) and weekly activity interference scores (AIS7 score) (both ranging from 0–21) were also calculated [22,23]. Higher scores reflected higher urticaria activity and greater interference with sleep and activities. 

The patients also recorded their DLQI scores at baseline and every 4 weeks. The DLQI has a recall period of 7 days and includes 10 questions covering six domains (symptoms and feelings, daily activities, leisure, work and school, personal relationships, and treatment). DLQI scores range from 0–30 and are interpreted using bands related to the impairment on a patient’s life (0–1 = no effect, 2–5 = small effect, 6–10 = moderate effect, 11–20 = very large effect, and 21–30 = extremely large effect). A higher DLQI score reflects a higher impairment of dermatology-related QoL [25,26], with a score of >10 generally indicating that a patient’s life is severely affected by their skin disease [26,27]. 

The impact of CSU on work among the employed patients was assessed every 4 weeks using the work productivity and activity impairment-chronic urticaria (WPAI-CU; version 2.0) questionnaire with a 7-day recall period [28]. The six-item WPAI-CU has four subscales, including absenteeism, presenteeism, and OWI (a composite score of absenteeism and presenteeism) for employed patients and activity impairment for all patients. The subscales are expressed as a percentage of impairment (0–100%). A higher score on the WPAI-CU reflects higher absenteeism and greater work and activity impairment.

### 2.3. Statistical Analysis

This analysis used the pooled data from all the treatment arms of the Phase 2b study (ligelizumab, omalizumab, and placebo) from baseline up to week 32. The percentages of PROM evaluations corresponding to DLQI scores of 0–1, an SIS7 score of 0, an AIS7 score of 0, and an OWI score of 0 for each UAS7 band were recorded.

The percentages of complete response PROM evaluations at consecutive UAS7 bands were compared using odds ratios (ORs). The ORs, 95% confidence intervals (CIs), and nominal *p*-value estimates were determined using generalised estimating equations with the UAS7 responder status and visit as the covariates. Least square means (LS), standard errors (SE), and nominal p-values were derived from the analysis of covariance (ANOVA) model, with visit, age, sex, and duration of CSU as the fixed effect factors and subject id as a random effect. SAS version 9.4 was used for these analyses.

## 3. Results

### 3.1. Baseline Demographics and Characteristics

Overall, 382 patients were included in this pooled analysis (Table 1). Of these patients, 75% were female, and the mean age was 43.3 years (±a standard deviation (SD) of 12.5). The mean ± SD duration of CSU was 4.3 ± 6.0 years, the mean time to diagnosis was 4.3 ± 6.0 years, and the UAS7 at baseline ranged from 28.6 to 31.7. In total, 67.0% of patients had severe disease activity and 31.2% had moderate activity, and 1.8% had an unknown UAS7. The overall mean ± SD DLQI score at baseline was 13.6 ± 7.2, and 58.6% of patients had a DLQI of >10 [26,27]. Of the DLQI subdomains, ‘symptoms and feelings’ and ‘daily activities’ were the two most affected (Figure S1). The overall mean ± SD SIS7 score at baseline for all patients was 10.6 ± 5.5, the overall AIS7 score was 11.1 ± 5.2, and the overall OWI score was 7.3 ± 17.5.

### 3.2. Complete Response to Treatment Is Linked to No Impact on DLQI

**Figure 1:**
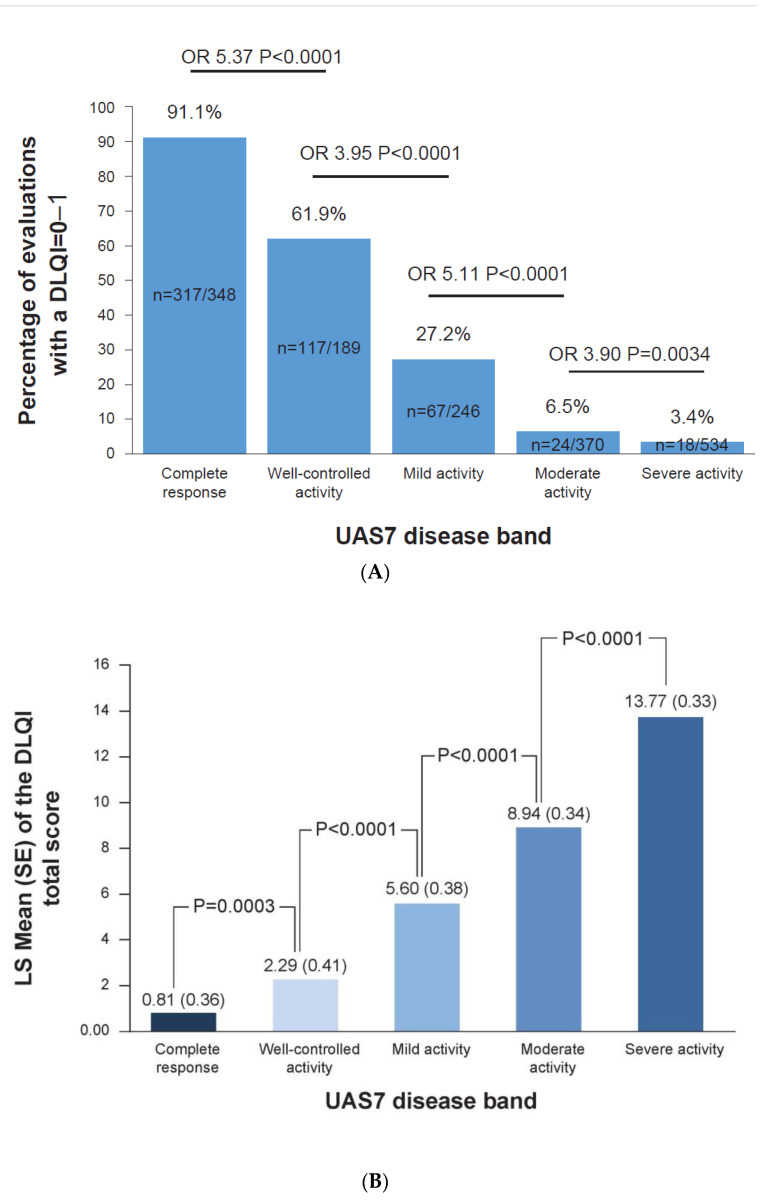
(**A**) Percentage of evaluations with a DLQI score of 0–1 in each UAS7 band. (**B**) DLQI scores for each UAS7 band. (**A**) Data up to week 32 from the Phase 2b core study, including baseline results, were included in this analysis. (**B**) Data up to week 32 from the Phase 2b core study, including baseline results, were included in this analysis. A DLQI = 0–1 score of 0–1 equated to no effect on a patient’s quality of life. UAS7 disease activity categories: complete response (UAS7 = 0), well-controlled activity (UAS7 = 1–6), mild activity (UAS7 = 7–15), moderate activity (UAS7 = 16–27), and severe activity (UAS7 = 28–42). DLQI, Dermatology Life Quality Index; LS, least squares; SE, standard error; n, number of associated evaluations; OR, odds ratio; UAS7, weekly Urticaria Activity Score.

In total, 91.1% of the UAS7 = 0 evaluations had a corresponding DLQI = 0–1 evaluation compared to only 61.9% of the UAS7 = 1–6 evaluations (odds ratio (OR) of UAS7 = 0 vs. UAS7 = 1–6 corresponding to DLQI = 0–1: 5.37, 95% CI: 3.16–9.14; *p* < 0.0001, Figure 1A). The least square (LS) mean total DLQI score for patients with complete responses versus well-controlled activity evaluations was 0.81 versus 2.29 (standard error (SE): 0.36; *p* = 0.0003), and each successive increase in the UAS7 severity band resulted in a statistically significant increase in the DLQI score (Figure 1B). 

### 3.3. Complete Response to Treatment Is Linked to No Impact on Sleep

**Figure 2:**
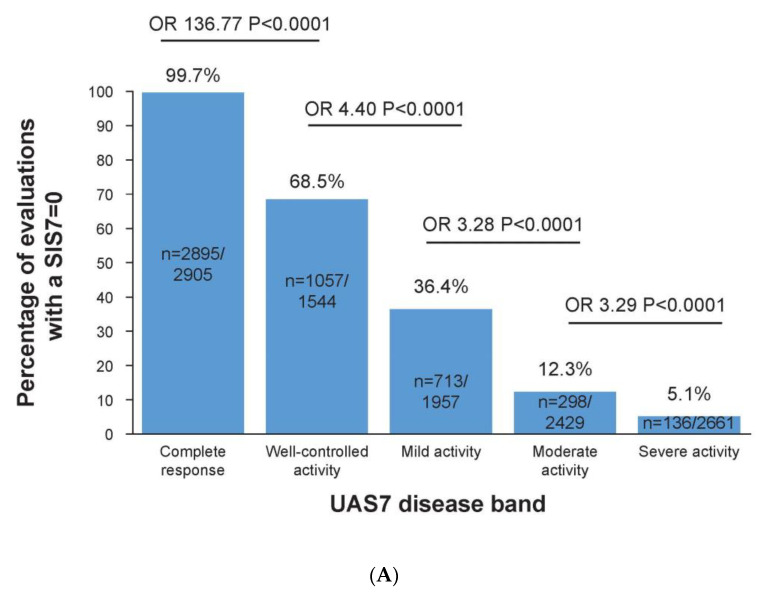

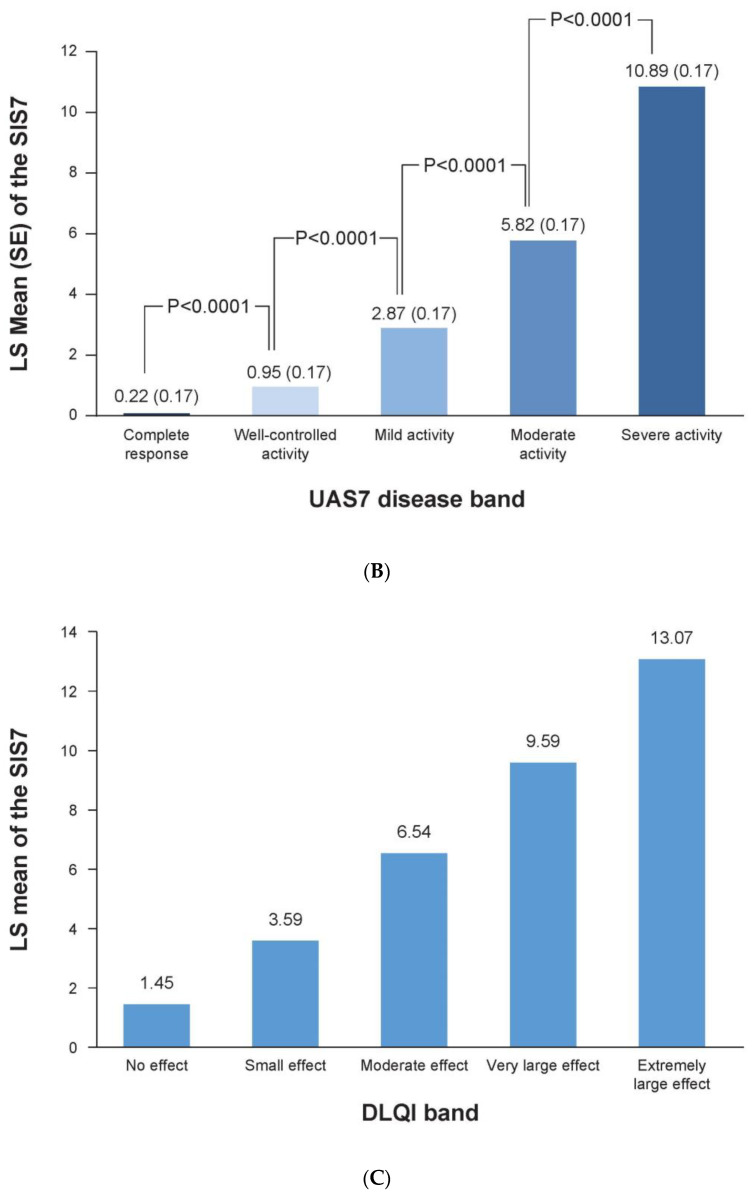
(**A**) Percentages of evaluations with an SIS7 of 0 in each UAS7 band. (**B**) SIS7 score for each UAS7 disease state. (**C**) The association between the SIS7 and DLQI bands. (**A**) SIS7 score by UAS7 disease band. SIS7 = 0 equated to no effect on a patient’s sleep. (**B**) Data up to week 32 from the Phase 2b core study, including baseline results, were included in this analysis. (**C**) SIS7 by DLQI band. DLQI bands: 0–1, no effect; 2–5, small effect; 6–10, moderate effect; 11–20, very large effect; and 21–30, extremely large effect. UAS7 disease activity categories: complete response (UAS7 = 0), well-controlled activity (UAS7 = 1–6), mild activity (UAS7 = 7–15), moderate activity (UAS7 = 16–27), and severe activity (UAS7 = 28–42). LS, least squares; N, number of associated evaluations; OR, odds ratio; SE, standard error; SIS7, weekly Sleep Interference Score; UAS7, weekly Urticaria Activity Score.

Almost all UAS7 = 0 evaluations (99.7%) had SIS7 = 0 evaluations compared to only 68.5% of UAS7 = 1–6 evaluations (OR of UAS7 = 0 vs. UAS7 = 1–6 corresponding to SIS7 = 0: 136.77, 95% CI: 37.20–502.83; *p* < 0.0001; Figure 2A). The SIS7 approximately doubled between each subsequent increase in the UAS7 disease activity band, and the differences between the successive bands were significant (*p* < 0.0001). The LS mean total for the SIS7 in patients achieving UAS7 = 0 versus patients with UAS7 = 1–6 was 0.22 versus 0.95 (SE: 0.17; *p* < 0.0001; Figure 2B). 

### 3.4. No Impairment of CSU on Life Is Linked to No Impact on Sleep and Daily Activities

**Figure 3:**
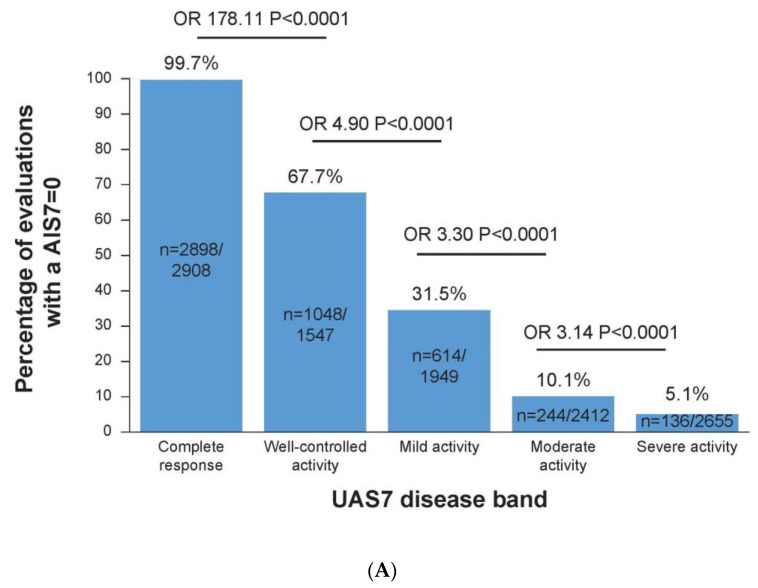

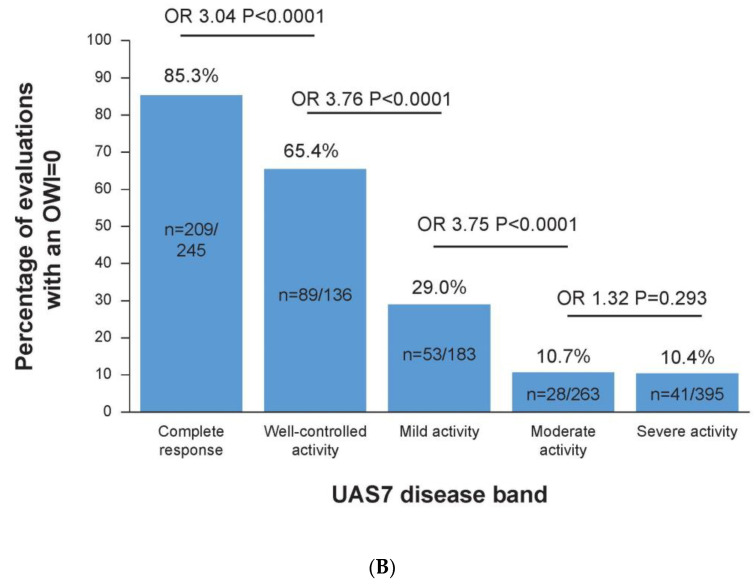
(**A**) Percentages of evaluations with AIS7 of zero in each UAS7 band. (**B**) Percentages of evaluations with an OWI of zero in each UAS7 band. Data up to week 32 from the Phase 2b core study, including baseline results, were included in this analysis. An AIS7 = 0 equated to no effect on a patient’s activity. An OWI = 0 equated to no effect on a patient’s work. UAS7 disease activity categories: complete response (UAS7 = 0), well-controlled activity (UAS7 = 1–6), mild activity (UAS7 = 7–15), moderate activity (UAS7 = 16–27), and severe activity (UAS7 = 28–42). AIS7, weekly Activity Interference Score; n, number of associated evaluations; OR, odds ratio; OWI, overall work impairment; UAS7, weekly Urticaria Activity Score.

Similarly, there was a clear association between the DLQI score and the SIS7 (Figure 2C); overall, a DLQI = 0–1 evaluation was associated with a low mean ± SE SIS7 evaluation of 1.45 ± 0.23. As the DLQI scores increased to reflect an increasing impairment of CSU on patients’ dermatology-related QoL scores, the associated SIS7 evaluations also increased (indicating more sleep impairment), and a DLQI of 2–5 corresponded to a mean ± SD SIS7 of 3.59 ± 0.25, a DLQI of 6–10 corresponded to an SIS7 of 6.54 ± 0.26, a DLQI of 11–20 corresponded to an SIS7 of 9.59 ± 0.25, and a DLQI of 21–30 corresponded to an SIS7 of 13.07 ± 0.35 (Figure 2C). Among the UAS7 = 0 evaluations at any time during the study, 99.7% of the concurrent AIS7 measurements were also equal to zero. In comparison, among the evaluations of UAS7 1–6 at any time during the study, 67.7% also had a concurrent evaluation of AIS7 = 0 (OR vs. UAS7 = 0: 178.11, 95% CI: 29.22–1085.8; *p* < 0.0001; Figure 3A).

### 3.5. Complete Response to Treatment Is Linked to No Impact on a Patients’ Work

Throughout the study, UAS7 = 0 evaluations had a clear association with OWI = 0 evaluations. A total of 85.3% versus 65.4% of evaluations achieved OWI scores of 0 in the UAS7 = 0 versus UAS7 1–6 band (OR: 3.04, 95% CI: 1.83–5.05; *p* < 0.0001, Figure 3B).

## 4. Discussion

Validated PROMs evaluating various aspects of CSU and its impacts on patients are important measures that help assess clinical changes over time and the effectiveness of treatments. Different PROMs assess different aspects of CSU, including disease activity, impact, and control. Although these are linked, they are independently important. Although a reduction in disease activity leads to improved control, control also leads to increased daily activities which may raise the risk of exacerbation. Thus, even though UAS7 may be low, control may not always be achieved. This is, indeed, evident from the results of this study, where the achievement of UAS7 scores of one to six resulted in a large reduction in patients achieving complete control of other PROMs compared with the patients who achieved UAS7 scores of zero. Likewise, high disease activity is linked with high burden, and a reduction in the former comes with a reduction in the latter but with different kinetics. Thus, it is important to assess patients for these three features of CSU using different PROMs in clinical practice.

The UAS7 is a validated, reliable, and widely accepted tool to assess CSU disease activity. In this analysis, achieving a complete response to treatment for itching and hives (i.e., a UAS7 value of zero), led to significant improvements in overall HRQoL, sleep quality, daily activities, and work productivity measurements. This was in line with data from a ligelizumab extension study where significantly better sleep was reported when the UAS7 score was low [29].

In this study, dermatology-related QoL and sleep parameters had a clear association with UAS7 disease activity bands, with a significantly higher percentage of DLQI = 0 and SIS7 = 0 evaluations (indicating no impairment on dermatology-QoL and sleep, respectively) being associated with a UAS7 = 0 evaluation compared to a well-controlled activity evaluation (UAS7 = 1–6). Well-controlled activity evaluations were associated with a much higher impact on HRQoL, as indicated by the lower percentage of evaluations corresponding to a DLQI = 0 and an SIS7 = 0, and the scores were progressively worse between each adjacent worsening disease activity band. A significant difference was observed in the evaluations for all PROMs between each adjacent UAS7 disease activity band, except for the OWI evaluations (there was no significance between the moderate (UAS7 = 16–27) and severe (UAS7 = 28–42) bands). These results emphasised the importance of aiming for complete disease activity control or a complete response to treatment. 

The results of this study were in line with recent publications that suggested that the HRQoL scores of patients with CSU are highly impacted by fatigue and considerably affect sleep quality and daily activities [4,30,31]. The results also aligned with those from a pooled analysis of three CSU randomised controlled clinical trials [17]. The pooled analysis showed significant differences in the DLQI scores, the chronic urticaria QoL questionnaire scores, sleep interference measurements, and daily activity interference measurements between adjacent UAS7 bands, and it recommended the use of five categorical UAS7 disease states in clinical practice [17]. Additionally, the present analysis showed that sleep interference was strongly correlated with DLQI status, and significantly better sleep was reported between each adjacent DLQI band, with the lowest impact on sleep observed in the DLQI = 0–1 band. The association of sleep interference with DLQI was also shown in the A World-wide Antihistamine-Refractory chronic urticaria patient Evaluation (AWARE) study [30]. Furthermore, the evaluations of no sleep interference corresponded with low activity interference scores, indicating an association between these two parameters. 

In the current analysis, better control of urticaria activity resulted in better control of all outcomes assessed by the PROMs, and a disease state of UAS7 = 0 was consistently associated with significantly better values in all PROMs. Although the conclusions here would benefit from further larger population-based studies to validate them, they substantiate the current international EAACI/GA^2^LEN/EuroGuiDerm/APAAACI guideline for urticaria, which recommends aiming to achieve complete symptom relief and disease control when treating patients with urticaria [18]. A real-world evidence registry has shown that the level of control of urticaria activity is measured more accurately with PROMs than with a physician’s global assessment [32]. A marked misalignment in how patients with CSU and their physicians experience disease control and treatment response was demonstrated; physicians often overestimate the benefit that patients gain from their treatment [32], and this is likely to contribute to the undertreatment of CSU and the failure to achieve complete control of CSU. Furthermore, the fluctuating nature of CSU may influence a physician’s assessment depending on the patient’s disease state at the time of the assessment, which could result in the prescription of unsuitable and ineffective treatment. All the results reported in the current analysis were from the PROMs, and they are therefore the most reliable source of measurement when considering the level of control over a patient’s disease. 

The results of this analysis highlight the importance and usefulness of integrating PROMs into routine clinical practice to improve the management of urticaria and achieve complete control. Such routine clinical practices would also help to achieve the Global Allergy and Asthma European Network’s aim of providing excellence in urticaria management globally [33]. To facilitate this endeavour further, the Urticaria Centers of Reference and Excellence Chronic Urticaria Self Evaluation application (CRUSE^®^ APP), which is available globally, uses the UAS7, the urticaria control test, and the Chronic Urticaria Quality of Life questionnaire PROMs to help patients with chronic urticaria track and manage their condition, together with their physician. A recent study showed that over half of patients with urticaria were very-to-extremely interested in using an app to monitor their disease activity [34].

The Phase 2b clinical trial had some limitations in relation to this analysis. The trial was designed to assess a dose-response for ligelizumab and was not powered for a correlation analysis; therefore, all analyses presented here are post hoc and exploratory. Additionally, the patients in this trial could have had a more severe form of urticaria than the average CSU patient, although theoretically, this would impact all groups under comparison equally. Finally, the conclusions of this study were based on data from the ligelizumab Phase 2b clinical trial, and although it had the advantage over real world studies of being prospective, blinded, and multicentric, further studies using larger and more diverse patient populations are needed to assess the reproducibility of these findings.

## 5. Conclusions

In conclusion, a complete response to treatment on itching and hives results in a lower impact of CSU on the HRQoL scores of patients, as reflected by the corresponding DLQI, SIS7, AIS7, and OWI scores. These results reinforce the guideline-recommended approach to treat patients with CSU until complete control of the disease is achieved [18].

## Figures and Tables

**Table 1:** Demographic and clinical characteristics of the patients with CSU in the Phase 2b ligelizumab study.

Characteristics	Total (N = 382) ^†^
Age, years ± SD	43.3 ± 12.5
Female, n (%)	286 (75)
BMI, kg/m^2^ ± SD ^‡^	27.91 ± 6.5
Race, n (%) ^§^	
Native American	1 (0.3)
Asian	76 (20)
Black	8 (2)
White	283 (74)
Other	12 (3)
IgE level, IU/mL	
Median	87.2
Range	0–14, 100
Weekly itch severity score ± SD ^¶^	13.1 ± 4.1
Weekly hives severity score ± SD ^¶^	17.3 ± 4.4
UAS7 ± SD ^††^	30.4 ± 7.4
Positive CU Index n (%) ^‡‡^	145 (38)
Mean duration of CSU, years ± SD	4.3 ± 6.0
Background medication, n (%)	
Locally approved dose of H_1_-antihistamine	164 (43)
Escalated dose of locally approved H_1_-antihistamine	218 (57)

BMI, body mass index; CSU, chronic spontaneous urticaria; CU, chronic urticaria; IgE, immunoglobulin E; UAS7, urticaria activity severity score; N, number of patients; n, number of patients in sub group; SD, standard deviation. ^†^–the study total included patients that were pooled from all treatment arms in the Phase 2b study (ligelizumab 24 mg (n = 43), ligelizumab 72 mg (n = 84), ligelizumab 240 mg (n = 85); omalizumab 300 mg (n = 85), placebo (n = 43), and ligelizumab 120 mg (single dose arm, n = 42); the 120 mg single-dose arm was chosen to characterise the pharmacokinetics/pharmacodynamics). ^‡^–the BMI was the weight in kilograms divided by the square of the height in metres. ^§^–race was reported by the patient or determined by the investigator. ^¶^–the weekly itch severity and hives severity scores measured severity over a period of 7 days on scales ranging from 0–21, with higher scores indicating greater severity. ^††^–the UAS7 was a composite of the weekly itch severity and hives severity scores, and the scores ranged from 0–42, with a higher score indicating greater severity. ^‡‡^–a positive CU index (the scores ranged from 1–50, with a score of ≥10 representing a positive result) indicated that the patient had either an autoimmune basis for the urticaria or an alternative histamine-releasing factor that was associated with greater disease severity than that in patients with a negative CU index.

## Data Availability

The data that support the findings of this study are available from the corresponding author upon reasonable request.

## Supplementary Materials

The following supporting information can be downloaded at: https://www.mdpi.com/article/10.3390/jcm12103561/s1, Figure S1: DLQI subdomain scores at baseline.
