# Supplementary material for: Why a Complete Response Is the Treatment Aim in Chronic Spontaneous Urticaria
Source: J Clin Med. 2023 May 19;12(10):3561. doi: 10.3390/jcm12103561 (PMC10219198; doi:10.3390/jcm12103561)
Supplement: Supplementary file 1 [file jcm-12-03561-s001.zip › jcm-2309316-supplementary.pdf]

Supplementary Figure S1

DLQI subdomain scores at baseline

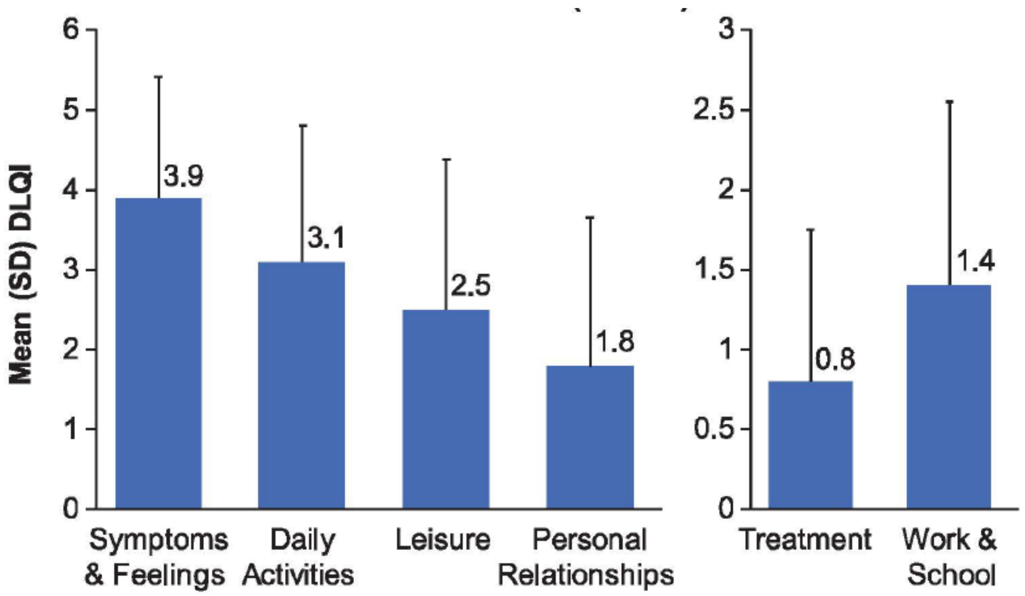

Supplementary Figure S1. The six subdomains of the DLQI were reported by patients at baseline. Bars indicate the SD. DLQI, dermatology life quality index; SD, standard deviation
